# Variation in Genome-Wide Levels of Meiotic Recombination Is Established at the Onset of Prophase in Mammalian Males

**DOI:** 10.1371/journal.pgen.1004125

**Published:** 2014-01-30

**Authors:** Brian Baier, Patricia Hunt, Karl W. Broman, Terry Hassold

**Affiliations:** 1 School of Molecular Biosciences, Washington State University, Pullman, Washington, United States of America; 2 Department of Biostatistics and Medical Informatics, University of Wisconsin-Madison, Madison, Wisconsin, United States of America; University of Chicago, United States of America

## Abstract

Segregation of chromosomes during the first meiotic division relies on crossovers established during prophase. Although crossovers are strictly regulated so that at least one occurs per chromosome, individual variation in crossover levels is not uncommon. In an analysis of different inbred strains of male mice, we identified among-strain variation in the number of foci for the crossover-associated protein MLH1. We report studies of strains with “low” (CAST/EiJ), “medium” (C3H/HeJ), and “high” (C57BL/6J) genome-wide MLH1 values to define factors responsible for this variation. We utilized immunofluorescence to analyze the number and distribution of proteins that function at different stages in the recombination pathway: RAD51 and DMC1, strand invasion proteins acting shortly after double-strand break (DSB) formation, MSH4, part of the complex stabilizing double Holliday junctions, and the Bloom helicase BLM, thought to have anti-crossover activity. For each protein, we identified strain-specific differences that mirrored the results for MLH1; i.e., CAST/EiJ mice had the lowest values, C3H/HeJ mice intermediate values, and C57BL/6J mice the highest values. This indicates that differences in the numbers of DSBs (as identified by RAD51 and DMC1) are translated into differences in the number of crossovers, suggesting that variation in crossover levels is established by the time of DSB formation. However, DSBs per se are unlikely to be the primary determinant, since allelic variation for the DSB-inducing locus *Spo11* resulted in differences in the numbers of DSBs but not the number of MLH1 foci. Instead, chromatin conformation appears to be a more important contributor, since analysis of synaptonemal complex length and DNA loop size also identified consistent strain-specific differences; i.e., crossover frequency increased with synaptonemal complex length and was inversely related to chromatin loop size. This indicates a relationship between recombination and chromatin compaction that may develop as DSBs form or earlier during establishment of the meiotic axis.

## Author Summary

During prophase of meiosis, homologous chromosomes exchange genetic material, in a process known as crossing-over. Crossovers are thought to be essential for proper separation of chromosomes during meiosis but, surprisingly, most mammalian species exhibit substantial individual variation in the number of crossovers per cell. We investigated the basis for this variation by examining localization patterns of crossover-associated proteins in inbred strains of male mice with differing average numbers of crossovers per spermatocyte. Our results indicate that the strain-specific variation is established early in meiotic prophase, possibly even before the DNA is broken in advance of subsequent exchanges between homologous chromosomes.

## Introduction

Recombination is a defining event of meiosis, resulting in the physical exchange of DNA between homologous chromosomes. It is generally thought that this is essential for proper alignment and subsequent segregation of homologs during meiosis I and, indeed, evidence from yeast [1], [2], *Caenorhabditis elegans* [3], *Drosophila melanogaster* [4], and mammals [5] indicates that alterations in the number or positioning of recombination events increase the likelihood of meiotic nondisjunction. For this reason, it might be expected that recombination levels are strictly regulated but, surprisingly, substantial inter-individual variation in recombination is observed in most mammalian species. For example, linkage studies have demonstrated extensive variation in recombination rates and/or recombination hotspot usage in human males and females (e.g., [6], [7], [8]), and cytological studies of recombination indicate 15–25% individual differences in genome-wide recombination levels in rhesus and human males [9], [10], and similar levels of variation in different inbred strains of mice [11], [12]. Further, the frequency and location of exchanges vary between the sexes; e.g., in humans the female genetic map is approximately 1.6 fold longer than that of males, with interstitial exchanges being more common in females than males (e.g., [13], [14], [15]). These differences beg an obvious question: what is responsible for the variation in recombination levels observed among individuals or inbred strains?

Arguably, there are at least three different time-points in the recombination process at which variation in recombination levels could arise. First, variation could be induced at the beginning of the recombination pathway, when double-strand breaks (DSBs) are formed. For example, some individuals might have a greater number of DSBs than others and, assuming that similar proportions of DSBs are converted into crossovers, the end result would be variation in the number of crossovers. On the surface, recent studies in mice would seem to eliminate this possibility: as in other model organisms [16], crossover homeostasis operates in male mice to ensure the presence of a minimum number of crossovers over a wide range of DSBs, suggesting that variation in DSBs does not translate into variation in crossovers [17]. However, these analyses were intended to assess variation in recombination among mice with different numbers of functional alleles for the DSB-inducing locus *Spo11*, and do not preclude the possibility that genetic background differences may influence the overall number of crossovers. Second, variation could arise at some stage in the processing of recombination intermediates. For example, genotypic differences at *RNF212*, encoding a protein with homology to crossover promoting proteins in *S. cerevisiae* and *C. elegans*, is the best characterized determinant of individual variation in genome-wide recombination rates in humans (e.g., [18]). Recently, analyses of mice deficient for RNF212 indicate that it acts downstream of the initiation of DSBs, stabilizing joint DNA molecules and promoting the resolution of DSBs as crossovers [19]. Taken together, these observations provide evidence that events occurring after the formation DSBs can, indeed, affect the eventual number of recombination events, although it is not clear that this accounts for all of the among-individual or among-strain differences that have been reported in mammals. Third, differences might arise at the end of the recombination pathway, owing to individual variation in proteins such as MLH1 and MLH3 that are important in the resolution of double Holliday junctions into crossovers (e.g., [20]).

To discriminate among these possibilities in a mammalian system, we took advantage of strain-specific differences in genome-wide rates of meiotic recombination in the male mouse [11]. Specifically, we used immunofluorescence to examine the localization patterns of early-, mid-, and late-acting meiotic recombination proteins, asking whether the patterns were the same or different among inbred strains known to have “low”, “mid”, or “high” genome-wide rates of recombination. For each strain, we analyzed the number of foci for RAD51 and DMC1 (strand invasion proteins acting shortly after double-strand break formation; [21]), MSH4 (part of the complex stabilizing double Holliday junctions; [22]), and BLM (thought to have anti-crossover activity; e.g., see [23]), and compared these observations with results of analyses of the CO-associated protein MLH1 [20].

Our results demonstrate that inter-strain differences in crossovers (MLH1 foci) are preceded by proportionally similar differences in early-acting recombination proteins, indicating that the variation is established at, or before, the time of DSB formation. Subsequent analyses of males heterozygous for the gene encoding the DSB-inducing protein SPO11 [24], allowed us to eliminate DSBs per se as the source of the variation, since *Spo11* heterozygotes exhibited a decrease in DSBs, but not in MLH1 foci.

In analyses of chromatin loop size and synaptonemal complex (SC) length, we detected striking differences among the three inbred strains, but not between *Spo11* heterozygotes and their wildtype littermates. Taken together with the observations on recombination proteins, our results suggest that strain-specific differences in chromatin architecture, presumably established prior to the initiation of recombination, are important determinants of variation in crossover frequency.

## Results

### Strain-specific variation in MLH1 distribution

**Figure 1:**
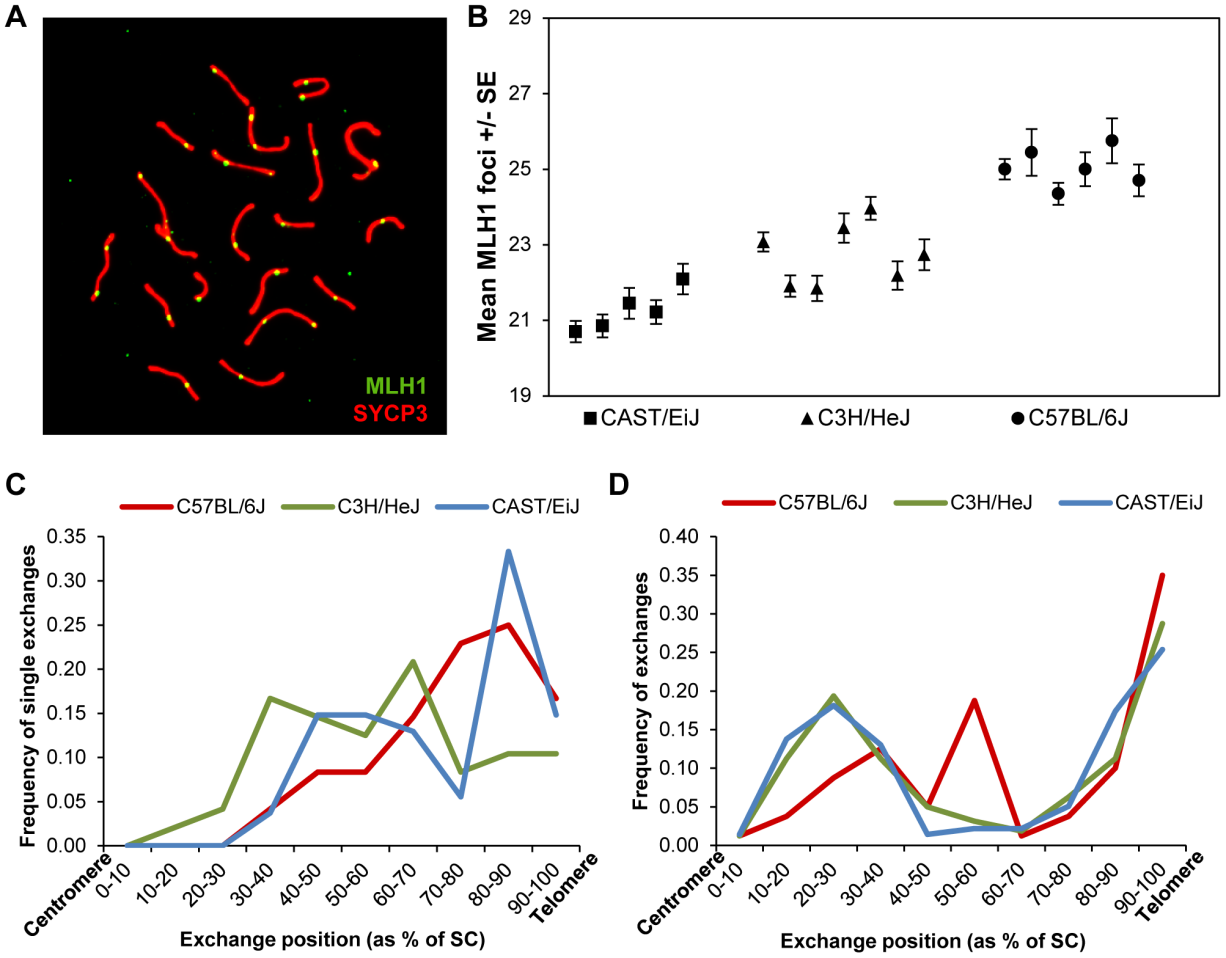
Inter-strain variation in mean MLH1 values. (**A**) Pachytene cell from B6 male immunostained with antibodies to MLH1 (green) and SYCP3 (red). The number of MLH1 foci per cell were counted and used as a surrogate for meiotic recombination events. (**B**) The mean number of MLH1 foci per spermatocyte for 5 CAST (n of cells = 105), 7 C3H (n of cells = 209) and six B6 (n of cells = 110) males varied significantly among the three inbred strains. (**C, D**) For each strain, slides were hybridized with FISH probes for chromosomes 19 and 1 and the positions of MLH1 foci were calculated as a percent of SC length (centromere = 0%; telomere = 100%). (**C**) On chromosomes 19 with a single MLH1 focus, the focus was typically medially or distally placed; data are based on analysis of 50 chromosomes/strain. (**D**) On chromosomes 1 with two MLH1 foci, typically one was proximally located and the other distally placed, consistent with positive interference. Data based on analysis of 50 cells/strain.

In previous studies of recombination in male mice [11], we identified strain-specific differences in the number of foci per cell of the DNA mismatch repair protein MLH1, known to mark the vast majority of sites of crossing-over [9], [26], [27]. We decided to exploit these differences to investigate the basis of the variation. Accordingly, we examined three inbred strains –C57BL/6J (“B6”), CAST/Ei (“CAST”) and C3H/HeJ (“C3H”) – assaying a minimum of 15 pachytene stage cells per mouse, and at least five mice per strain, scoring the number of autosomal MLH1 foci per cell (Figure 1A).

Two of the inbred strains, CAST and B6, had previously been found to have “low” and “high” genome-wide MLH1 values, respectively [11]. Our re-analysis produced virtually identical results: the mean number +/− S.D. of autosomal MLH1 foci per cell was 21.3+/−1.6 for CAST (n = 105 cells) and 25.0+/−2.2 for B6 (n = 102 cells) (Figure 1B; Table S1). Subsequently, we analyzed C3H males, and observed that this strain had mean MLH1 values that were intermediate to the other two strains: i.e., 22.7+/−1.9 (n = 209 cells) (Figure 1B; Table S1). Because the number of MLH1 foci per cell is not normally distributed (i.e., typically each bivalent has at least one focus, thus constraining the autosomal foci per cell to 19 or more), inter-strain differences have to be interpreted with caution. Nevertheless, ANOVA analyses are typically robust in the face of modest departures from normality, and the magnitude of the differences we observed (F = 105.1; p<0.0001), make it likely that the variation is real. Thus, these observations confirm our previous conclusion of variation in genome-wide MLH1 values – and presumably MLH1-driven crossovers – among males of different mouse strains.

The variation in overall MLH1 frequency was reflected by highly significant strain-specific differences in the proportion of chromosomes with zero, one, two, or three MLH1 foci (χ^2^ = 292.0, p<0.00001; Table S1). In large part, the difference was attributable to differing ratios of chromosomes with one vs. two MLH1 foci in the three strains; i.e., 6.8∶1, 3.9∶1 and 2.2∶1 for CAST, C3H and B6, respectively (Table S1). However, intriguingly, the strain with the highest MLH1 average value (B6), also had the highest proportion of bivalents lacking MLH1 foci; indeed, this value was significantly increased over that for CAST (χ^2^ = 12.1, p<0.001) and for C3H (χ^2^ = 13.9, p<0.001) males.

Subsequently, we analyzed the placement of MLH1 foci among the three strains, asking whether variation in MLH1 levels might be linked to differences in the location of the foci. Initially, we simply pooled results from all homologs and found no obvious differences among the strains; i.e., consistent with previous results, distally located MLH1 foci predominated among all three strains [11]. However, because the strain-specific differences in the proportion of homologs with one, two and three MLH1 foci complicate interpretations of these data, we conducted a second set of studies in which we analyzed individual chromosomes. Specifically, for each strain, we analyzed the placement of MLH1 foci on chromosomes 19 and 1 on SCs that exhibited one and two foci, respectively (Figure 1C, D). No obvious differences were observed among the strains.

### Localization patterns of proteins involved in repair of DSBs vary among strains

**Figure 2:**
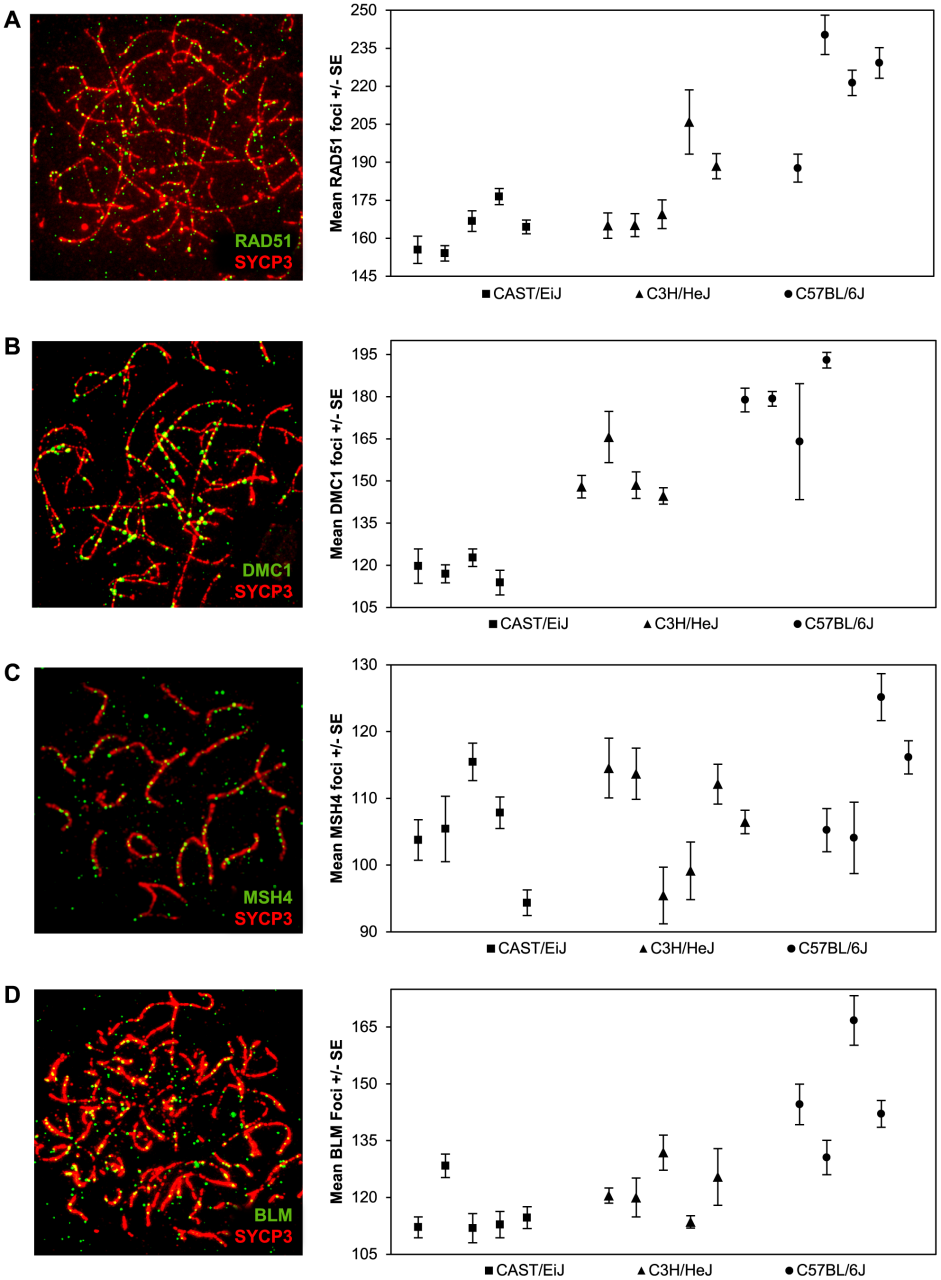
Inter-strain variation in early- and mid-stage meiotic recombination proteins. The number and location of foci for proteins acting upstream of MLH1 in the recombination pathway [i.e., (**A**) RAD51 (**B**) DMC1 (**C**) MSH4] or anti-recombination pathway [i.e., (**D**) BLM] were determined for each of the three strains and inter-strain values compared. For each of the four proteins, mean numbers of foci per cell varied significantly among the three strains. Data for each protein were based on 4–6 animals per strain, and a minimum of 60 cells per strain (see Supplemental Tables 2–5 for data).

The MLH1 data demonstrate inter-strain differences in recombination, but provide no information on when the variation originates. To address this question, we examined the abundance and distribution of signals for proteins involved at different stages of DSB repair, as follows:

Single end invasion is the earliest stage of recombination that can be consistently assayed using immunofluorescence. The ubiquitous RAD51 protein forms a complex around the resected ends of DSBs and facilitates invasion of the intact homologous chromosome [28]. We assayed localization patterns of RAD51 foci on zygotene stage chromosomes (Figure 2A) when RAD51 activity is high [29]; at this stage most RAD51 foci localize to synapsed regions, although in a few instances they (and also DMC1 foci, see below) are associated with unsynapsed axial elements. From Figure 2A, it is clear that there was considerable among-animal variation, particularly in the C3H and B6 strains. Nevertheless, considering the pooled data, the mean numbers of RAD51 foci per cell were highly significantly different among the three strains (F = 113.7; p<0.0001), with the variation mirroring that observed for MLH1; i.e., CAST had the lowest mean value (163.0+/−18.6), C3H an intermediate value (179.9+/−28.0), and B6 the highest value (222.1+/−33.8) (Figure 2A; Table S2). Further, considering those animals for which we had both RAD51 and MLH1 values (see Tables S1 and S2), the ratios of RAD51:MLH1 foci were similar among the three strains, with values of 7.7∶1 for CAST, 7.3∶1 for C3H and 8.8∶1 for B6.

Other than RAD51, relatively few animals were scored for both MLH1 and another recombination protein (i.e., either DMC1, MSH4 or BLM) in each of the three strains; thus, we were not able to directly compare the ratios of MLH1:DMC1, MLH1:MSH4 or MLH1:BLM among the three strains. Nevertheless, the pooled data were consistent with the results from MLH1 and RAD51. For example, similar strain-specific differences were observed for zygotene stage cells scored for the meiosis specific strand invasion protein DMC1, which attaches to DSB sites shortly after RAD51 [29], [30]. Specifically, the mean values rose from 119.5+/−16.4 for CAST to 149.3+/−18.1 for C3H and 181.8+/−21.1 for B6 (F = 214.3; p<0.0001) (Figure 2B; Table S3). Consistent with previous reports on DMC1 localization in mice [29], all three strains exhibited fewer DMC1 than RAD51 foci. While the reason for this variation in numbers of DMC1 and RAD51 foci is not entirely clear, it presumably reflects the different roles the two proteins play in the early stages of the recombination pathway [28], [31], [32] or possibly, differences in the time that the proteins remain complexed to the recombination nodules.

We investigated a later stage of recombination by assaying MSH4, a member of the MSH4-MSH5 complex that is thought to stabilize recombination intermediates (e.g., [19], [22], [33]. Since MSH4 localizes only to synapsed chromosome regions (Figure 2C), we counted foci in cells at the zygotene/pachytene boundary. We observed substantial variation in the values among the different individual mice but, similar to the results for RAD51 and DMC1, we saw an increase in the mean number of MSH4 foci from CAST (105.3+/−12.8) to C3H (109.0+/−14.8) and B6 (112.7+/−17.5) males (Figure 2C; Table S4). These differences were more modest than those identified for RAD51 and DMC1 and because there was considerable overlap in individual values from strain to strain, they need to be interpreted with caution. Nevertheless, the mean values for the “low” (CAST) and “high” (B6) strains were still highly significantly different from one another (t = 2.9; p<0.01); for CAST vs C3H and C3H vs B6 the values were non-significantly different (t = 1.5, p>0.13 and t = 1.5, p>0.14, respectively)

Finally, we analyzed zygotene cells for the helicase BLM, a regulator of recombination intermediates suggested to have anti-crossover activity ([23]; Figure 2D; Table S5). Strain specific differences in average numbers of BLM foci were similar to those observed for the positive regulators of crossovers; i.e., the mean value was lowest for CAST (114.4+/−15.9), intermediate for C3H (120.0+/−12.4), and highest for B6 (146.4+/−26.3) (F = 62.3; p<0.0001).

In summary, our observations on strain-specific variation for five different recombination pathway proteins (RAD51, DMC1, MSH4, BLM and MLH1) are consistent with one another; i.e., in each instance the mean number of foci per cell was lowest for CAST males, intermediate for C3H males, and highest for B6 males. Unfortunately, we were not able to collect data on each protein from each animal, limiting our ability to directly compare values among the different strains and, accordingly, to address other obvious questions, such as: which protein is the best predictor of MLH1 values, are the ratios of foci for different proteins the same among all three strains, and does the ratio of recombinogenic∶anti-recombinogenic proteins (e.g., RAD51:BLM) vary among strains? Nevertheless, taken together, the data provide strong evidence that, at least in males of these strains, a similar proportion of DSBs are translated into MLH1-associated crossovers.

### Variation in chromatin configuration is correlated with MLH1 levels

**Figure 3:**
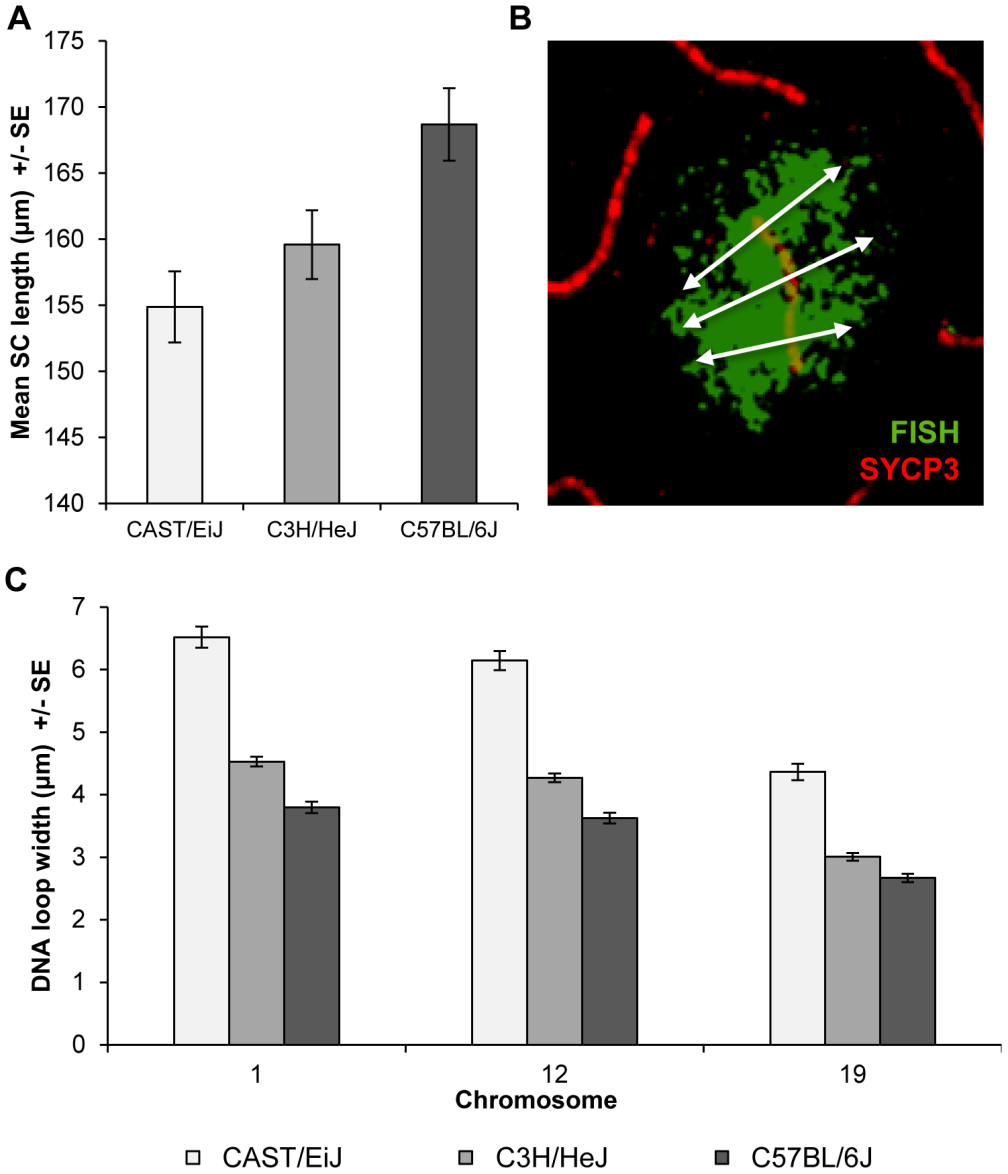
Inter-strain variation in SC length and DNA loop size. (**A**) Mean autosomal SC lengths varied in direct relationship to MLH1 values; i.e., the strain with the lowest mean MLH1 values (CAST) had the shortest SCs, while the strain with the highest MLH1 values (B6) had the longest SCs. Data are based on analyses of mid-pachytene spermatocytes from 2 CAST (n = 8 cells), 3 C3H (n = 11 cells) and 2 B6 (n = 21 cells) males. (**B**) DNA loop sizes for individual chromosomes were calculated as the average of the width of the FISH signal at three points along the SC: at the centromere, the midpoint, and the telomere; image shows example for chromosome 12. (**C**) Chromosomes 1, 12, and 19 were examined and for each, we observed an inverse relationship between strain-specific mean MLH1 values and mean DNA loop sizes.

In subsequent studies, we were interested in determining whether the strain differences were accompanied by variation in the configuration of the meiotic axis and/or DNA loops. Accordingly, for each strain we assayed total autosomal SC lengths in pachytene stage cells. We observed highly significant differences in mean SC length among the strains; i.e., for CAST 156.7+/−2.0 µm, for C3H 161.5+/−1.8 µm, and for B6 170.7+/−1.9 µm (F = 13.8; p<0.0001). These results are similar to the strain-specific observations for MLH1, suggesting that the strain differences in crossover levels are linked to variation in length of the meiotic axis (Figure 3A).

As a surrogate for DNA loop size, we examined the width of the signal from whole chromosome paint probes on representative large, medium-sized and small chromosomes; i.e., chromosomes, 1, 12 and 19, respectively (see Figure 3B for an example of a chromosome 12 paint probe). DNA loop sizes differed significantly among the strains for each of the three chromosomes: i.e., for chromosome 1, CAST = 6.5+/−1.3 µm, C3H = 4.5+/−0.6 µm and B6 = 3.8+/−0.7 µm (F = 183.5; p<0.0001); for chromosome 12, CAST = 6.2+/−1.2 µm, C3H = 4.3+/−0.6 µm and B6 = 3.6+/−0.7 µm (F = 176.5; p<0.0001); and for chromosome 19, CAST = 4.4+/−1.0 µm, C3H = 3.0+/−0.5 µm and B6 = 2.7+/−0.5 µm (F = 116.5; p<0.0001) (Figure 3C). Together with the observations on SC length, these analyses indicate that increasing MLH1 values are associated with smaller DNA loops and longer SCs.

### DSB number per se does not regulate MLH1 levels or chromatin morphology

The correlation between the number of foci for “early”, “mid”, and “late” recombination proteins and strain-specific recombination levels raises the possibility that the number of DSBs per se regulates recombination. We tested this by comparing meiotic profiles of males on the same genetic background but with different rates of DSB formation. Specifically, we compared wildtype B6 males with siblings heterozygous for a null allele of *Spo11*, the type II topoisomerase-like protein responsible for programmed DSB formation in meiocytes [24]. Notably, the results from the wildtype Spo11 males were somewhat different than those of the B6 male described above, presumably due to variation from maintenance of the stocks at different facilities.

**Figure 4:**
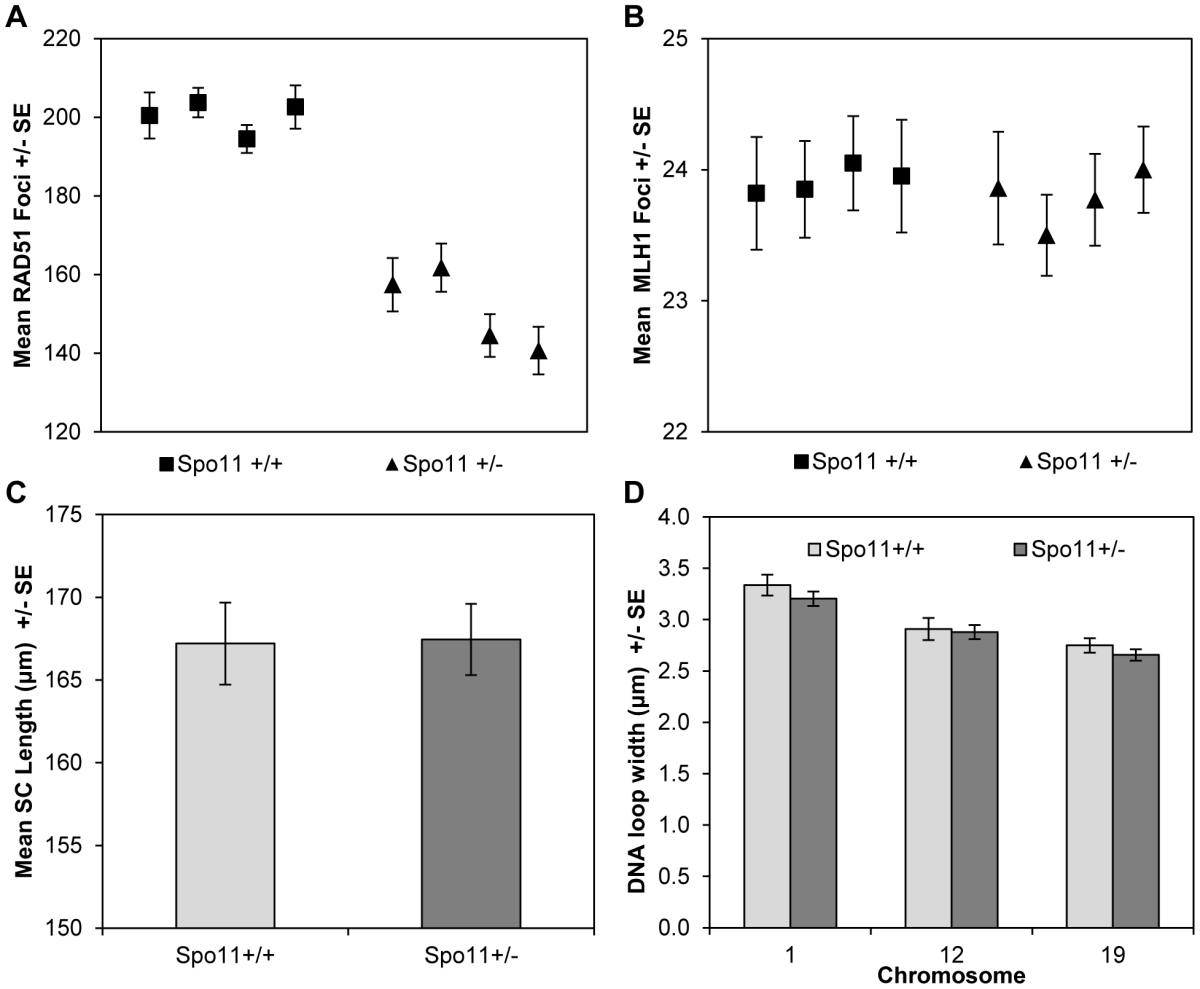
*Spo11^+/+^* and *Spo11^+/−^* animals. (**A**) Mice heterozygous for a null allele of *Spo11* exhibited a significant decrease in RAD51 foci (a marker of DSBs) by comparison to wildtype littermates. However, (**B**) the mean number of MLH1 foci (a marker of COs), (**C**) mean SC lengths and (**D**) mean DNA loop sizes were not different between the two genotypes.

For the *Spo11* animals, consistent with previous reports [34], [35], zygotene spermatocytes from *Spo11^+/−^* males displayed significantly fewer DSBs (estimated by the number of RAD51 foci) than spermatocytes from wildtype littermates (mean values = 152.2+/−20.6 and 200.5+/−21.5, respectively; t = 5.8, p<0.0001) (Figure 4A;Table S6). However, this did not translate into a difference in MLH1 values, with mean values of 23.9+/−1.9 and 23.8+/−1.6 for heterozygotes and wildtype littermates, respectively (Figure 4B; Table S7).

Although it altered RAD51 values, *Spo11* heterozygosity had no obvious effect on chromatin morphology. Specifically, total SC lengths per cell were virtually identical for *Spo11^+/+^* and *Spo11^+/−^* littermates (167.1+/−3.3 µm and 169.7+/−4.8 µm, respectively; t = 0.46 p>0.65) (Figure 4C). Further, there were no consistent differences in DNA loop size on individual chromosomes between *Spo11^+/+^* and *Spo11^+/−^* males [i.e, 3.3+/−0.5 µm and 3.2+/−0.5 µm, respectively, for chromosome 1 (t = 1.01, p = 0.31); 2.9+/−0.5 µm and 2.9+/−0.5 µm, respectively, for chromosome 12 (t = 0.27, p = 0.78), and 2.8+/−0.3 µm and 2.7+/−0.4 µm, respectively, for chromosome 19 (t = 1.00, p = 0.32)] (Figure 4D). Taken together with the results from the RAD51 and MLH1 assays, this indicates that the variation in crossover level among strains is not simply due to variation in the number of DSBs but more likely reflects differences in chromatin morphology.

## Discussion

The purpose of this study was to investigate the basis for individual variation in recombination rates. Over the past 10–15 years a number of tools have become available to investigate the biology of meiotic recombination in mammals; e.g., knockout mice have been used to identify and characterize the functions of numerous meiotic genes (e.g.,[20], [36], [37], [38], [39]); genotypic analysis of individual and pooled sperm samples has led to the identification of small, discrete regions of high recombination activity [40], [41], [42], [43]; linkage and linkage disequilibrium studies have revealed the presence of thousands of recombination hotspots in mammalian genomes [7], [44], [45]; and genome-wide analyses of genetic polymorphisms have led to the identification of genes involved in modulating hotspot activity [46], [47], [48] or genome-wide recombination levels [13], [18].

Nevertheless, our understanding of the origin of among-individual variation in recombination levels in mammals remains rudimentary. In recent studies, genotypic differences at three loci have been linked to variation in the recombination phenotype. Specifically, allelic variation in the gene encoding the meiosis-specific histone methytransferase PRDM9 has been associated with hotspot activity in both mice and humans [46], [47], [48]; allelic variation in *RNF212*, a homolog of the C. elegans synapsis/cross-over associated gene *zhp-3*, has been shown to affect genome-wide recombination levels in human males and females [13], [14], [18]; and the presence of an inversion at 17q21.31 affects recombination rates in human females [13], [14], [49]. However, several lines of evidence indicate that these individual loci may be relatively unimportant, or at least not the only, determinants of variation in CO levels. First, in studies of *PRDM9* in humans, allelic variation has been shown to influence hotspot usage but appears to have relatively little effect on genome-wide recombination levels [8], [46], [47], [50]. Second, the magnitude of the effects attributable to the other two loci (*RNF212* and inv17q21.31) is insufficient to account for the level of variation in genome-wide rates identified in humans or in the present study [13], [18]. Finally, in recent analyses of inbred strains of mice with differing levels of genome-wide recombination conducted by us [51] and others [52], putative QTL-containing chromosome regions did not include either *Prdm9* or *Rnf212*. Thus, the available evidence suggests that other, as yet unidentified, loci, are responsible for generating most of the variation in recombination rates among individuals or inbred strains.

To address this problem in a somewhat different way, we utilized inbred strains of mice with varying levels of genome-wide recombination to identify the temporal window during which the variation is generated. Surprisingly, our results provide strong evidence that the variation is attributable to processes acting at, or upstream of, DSB formation and repair. Specifically, our observations on foci number for each of three recombination-promoting proteins (RAD51, DMC1, and MSH4) and for the anti-recombination protein BLM were similar in finding a direct correlation between foci numbers and levels of recombination. In each of the three strains, the number of DSBs (measured as RAD51 foci) exceeded the number of crossovers (MLH1 foci) by approximately ten-fold. This is consistent with observations from previous studies of male mice (e.g, [53]) and indicates that as in other organisms, the vast majority of DSBs are repaired as non-crossovers. Further, it suggests that, at least for the inbred strains we examined, events occurring downstream of DSBs are relatively unimportant in mediating genetic background-dependent variation in recombination rates. This does not mean that these processes are irrelevant – e.g., *RNF212*, which is known to affect recombination levels in humans, has recently been show to stabilize proteins important in processing recombination intermediates in male mice [19] – only that there are other, more important determinants.

While our observations indicate that the variation in recombination levels is established by the time of DSB formation, our analyses of *Spo11* deficient animals suggest that this is not attributable to DSBs per se. That is, despite having significantly different numbers of RAD51 foci, wildtype and heterozygous animals had virtually identical mean MLH1 values. These results are similar to those recently reported by Cole and colleagues [17], in which they demonstrated a positive correlation between the number of functional *Spo11* genes and the number of DMC1 and RAD51 foci, but no accompanying change in the number of MLH1 foci. Thus, as in other model organisms (e.g., [16], [54]), mammalian meiosis appears to have homeostatic controls that operate to maintain the number of crossovers in the face of variation in the number of DSBs.

Taken together, our analyses of different inbred strains of mice and of mice varying at a single locus but on an otherwise isogenic background demonstrate that DSB number itself is not the driver of variation in CO levels. What other processes might be responsible? One obvious possibility is the way in which the chromatin is packaged, an idea consistent with the observations of the present report. For example, the best predictor of the number of MLH1 foci across the five different categories of mice that we examined (i.e., CAST, C3H, B6, *Spo11^+/+^* and *Spo11^+/−^*) was SC length, since in each instance there was an average of approximately 7 µm SC per MLH1 focus. Similarly, the relationship between DNA loop sizes on individual chromosomes and the number of MLH1 foci was comparable in animals of different genetic background or *Spo11* genotype. An obvious caveat to this interpretation is the fact that the observations on SC length and DNA loop size were based on pachytene cells while, presumably, the determination of the relative level of COs occurs much earlier. However, in unrelated studies of human males and females, we found that sex-specific differences in chromatin morphology in pachytene stage meiocytes were mirrored in leptotene preparations [55]. Thus, we think it unlikely that the observations of the present report simply reflect pachytene cells. Accordingly, we suggest that modification of chromatin morphology – but not DSB numbers – is a primary determinant of CO levels. This is consistent with recent analyses of Petukhova and Camerini-Otero and colleagues [56], [57], as they found that trimethylation of H3K4 at potential PRDM9 recombination hotspots is not dependent on SPO11; i.e., the establishment of hotspot-associated marks occurs regardless of the presence of DSBs.

What genetic loci might be at work to influence chromatin packaging and, ultimately, variation in CO levels? In two recent QTL analyses of recombination in different inbred strains of [51], [52], several QTLs were shared between the studies; i.e., overlapping regions were identified on chromosomes 4, 15 and 17, and nearly overlapping regions on chromosome 3 and the X chromosome. Notably, in both studies the highest lod scores were observed on the X chromosome, consistent with previous studies linking X-linked loci with variation in genome-wide recombination rates [12], [58]. Because inactivation of the sex chromosomes (MSCI) appears to be essential for normal male meiosis [59], it is generally thought that few, if any, X-linked loci are necessary for spermatogenesis. The results of the analyses of Murdoch et al [51] and Dumont and Payseur [52], as well as studies identifying spermatogenic functions for X-linked genes (e.g., *Tex11*; [60]), suggests that this is not an absolute rule, and that high resolution analyses of the X chromosome may uncover important recombination-associated loci.

## Materials and Methods

### Ethics statement

All animal experiments and procedures conducted at Washington State University were performed using protocols approved by the Institutional Animal Care and Use Committee (WSU IACUC number 03737-014).

### Mice

Breeding stocks of three inbred strains, C57BL/6J, C3H/HeJ, and CAST/EiJ, were obtained from The Jackson Laboratory and maintained by brother-sister mating. *Spo11^+/+^* and *Spo11^+/−^* animals (on a C57BL/6J background) were kindly provided by Drs. Maria Jasin and Scott Keeney, Memorial Sloan Kettering Cancer Center. All animals were housed in polysulfone cages on ventilated racks or static cages. Experiments were approved by the Washington State University (WSU) Institutional Animal Care and Use Committee. WSU is fully accredited by the American Association for Accreditation of Laboratory Animal Care and all investigations were conducted in accordance with the Guide for the Care and Use of Laboratory Animals.

### Tissue collection

Adult male mice between the ages of 6 and 40 weeks were killed by cervical dislocation and the testes removed. Seminiferous tubules were removed from the testes and surface spread preparations of spermatocytes for immunofluorescence studies prepared as described previously [61].

### Immunostaining

Slides were immunostained using similar methodology to that of Anderson *et al.* [26]. Antibodies were diluted in sterile filtered 1×ADB consisting of 10 ml normal donkey serum (Jackson ImmunoResearch), 3 g BSA (Sigma-Aldrich), 50 µl Triton X-100 (Alfa Aesar), and 990 ml PBS. Incubations were performed in a dark humid chamber at 37°C.

Slides were incubated overnight in a dark humid chamber at 37°C with one of the following primary antibodies: RAD51 (rabbit anti-human, Santa Cruz Biotechnology) diluted 1∶75, DMC1 (rabbit anti-human, Santa Cruz Biotechnology) diluted 1∶200, BLM (rabbit anti-human, Santa Cruz Biotechnology) diluted 1∶100, MSH4 (rabbit anti-human, provided by Dr. Chengtao Her) diluted 1∶75, or MLH1 (rabbit anti-human, Calbiochem) diluted 1∶75. All slides were incubated with a primary antibody for SYCP3 (goat anti-mouse, Santa Cruz Biotechnology) diluted 1∶100 for 2 hours. Fluorescein-labeled donkey anti-rabbit (Jackson ImmunoResearch) secondary antibody was diluted 1∶75 and incubated overnight, followed by an incubation for 45 minutes with rhodamine-labeled donkey anti-goat secondary antibodies (Jackson ImmunoResearch), diluted 1∶200. Slides were counterstained and fixed using Prolong Gold antifade reagent with DAPI (Invitrogen) and sealed with rubber cement.

### Fluorescence in-situ hybridization

StarFish whole chromosome paint probes (Cambio) were used according to the manufacturer's instructions to estimate DNA loops of specific chromosomes. Briefly, previously immunostained slides were dehydrated in a series of brief ethanol washes (75%, 90%, 100%), denatured in a 70% dionized formamide/2×SSC solution for 5 minutes at 65°C, quenched in 70% ethanol at −20°C, and dehydrated again with a series of ethanol washes. Paint probes for chromosomes 1, 12, and 19 were denatured for 10 minutes at 65°C and applied to the slides overnight in a dark humid chamber at 37°C. Following incubation, slides were soaked twice for 5 minutes in 50% dionized formamide/1×SSC solution at 45°C, washed twice in 1×SSC at 45°C for 5 minutes, and soaked for three cycles in 25% Detergent DT/4×SSC (Cambio) for 4 minutes at 45°C. Slides were allowed to drain and mounted with Reagent MD (Cambio) and sealed with rubber cement.

### Microscopy and scoring

For immunofluorescence analysis, cells were identified using DAPI and the sub-stage of meiotic prophase determined on the basis of the morphology of the SC (visualized with SYCP3 antibody). For each cell and each protein analyzed, we scored the number of foci localizing to the axial element or SC at the appropriate sub-stage of meiotic prophase; i.e., for RAD51, DMC1, and BLM prior to complete synapsis during zygotene, MSH4 at the zygotene-pachytene transition, and MLH1 during pachytene prior to desynapsis of the XY bivalent. For analysis of MLH1 foci we restricted our scoring to autosomal chromosomes, since the appearance and disappearance of foci on the XY bivalent and on the autosomes are temporally uncoupled.

Position data for MLH1 foci was collected by measuring along the fully synapsed SC from the centromere to each MLH1 focus. The placement of MLH1 was calculated by dividing the centromere-MLH1 distances by the full length of each individual SC. Values are represented as a percentage of total SC length, with 0% being the centromeric end and 100% the telomeric end of the SC.

Genome-wide SC length was measured in fully synapsed pachytene cells by manually tracing the length of the SYCP3 signal for all SCs except the XY bivalent. The genome-wide SC length per cell was calculated as the sum of the SYCP3 signal lengths for the 19 autosomes.

DNA loop sizes were assayed on three representative chromosomes, 1, 12, and 19, identified by FISH paint probes, using an approach similar to that previously described by Novak et al [62] FISH images were then overlaid on immunofluorescence images of the SC and loop. Loop size was assayed by taking linear measurements of the width of the FISH signal perpendicular to the SC at the centromere, telomere, and mid-point of the SC. The three lengths for each chromosome were averaged and compared on a chromosome-specific basis among the strains.

All slides were imaged on a Zeiss Axio Imager epifluorescence microscope and analyzed by blinded observers using Zeiss Axiovision software (version 4.7).

### Statistical analysis

Comparisons in the average numbers of foci between different strains or genotypes of mice were tested by analysis of variance or Student t-test analysis, pooling the results from multiple mice in a strain or genotype. For post-hoc comparisons of mean values between specific strains, we used the Bonferroni correction to account for the multiple tests. Mean values +/− SD are provided in the text and tables and, for illustrative purposes, as mean values +/− SE in the figures. Differences in the numbers of E0 bivalents were compared by chi-square tests. All statistical analyses were performed with JMP software, version 7.0.1 (SAS Institute Inc.).

## Supporting Information

Table S1 Mean +/− S.D. MLH1 foci numbers for each animal and inbred strain. Significant strain-specific differences were evident, with B6 having the highest mean values, C3H intermediate values and CAST the lowest values. Similar strain-specific differences were observed for other recombination pathway proteins, and are summarized below in Tables S2-S5.
(DOCX)

Table S2 Mean +/− S.D. RAD51 foci numbers for each animal and inbred strain.
(DOCX)

Table S3 Mean +/− S.D. DMC1 foci numbers for each animal and inbred strain.
(DOCX)

Table S4 Mean +/− S.D. MSH4 foci numbers for each animal and inbred strain.
(DOCX)

Table S5 Mean +/− S.D. BLM foci numbers for each animal and inbred strain.
(DOCX)

Table S6 Mean +/− S.D. RAD51 foci numbers for *Spo11^+/−^* and *Spo11^+/+^* males.
(DOCX)

Table S7 Mean +/− S.D. MLH1 foci numbers for *Spo11^+/−^* and *Spo11^+/+^* males.
(DOCX)
