# Supplementary material for: Variation in Genome-Wide Levels of Meiotic Recombination Is Established at the Onset of Prophase in Mammalian Males
Source: PLoS Genet. 2014 Jan 30;10(1):e1004125. doi: 10.1371/journal.pgen.1004125 (PMC3907295; doi:10.1371/journal.pgen.1004125)
Supplement: Table S1 — Mean +/− S.D. MLH1 foci numbers for each animal and inbred strain. Significant strain-specific differences were evident, with B6 having the highest mean values, C3H intermediate values and CAST the lowest values. Similar strain-specific differences were observed for other recombination pathway proteins, and are summarized below in Tables S2-S5. (DOCX) [file pgen.1004125.s001.docx]

Table S1: Mean +/- S.D. MLH1 foci numbers for each animal and inbred strain.

|  | **Mouse** | **MLH1 Ave +/- SD** | **No. of Cells** | **Range** | **E0*** | **E1*** | **E2*** | **E3*** |
| --- | --- | --- | --- | --- | --- | --- | --- | --- |
|  | CAST/EiJ 1 | 20.70 +/- 1.26 | 20 | 19-23 | 6 | 334 | 40 | 0 |
|  | CAST/EiJ 2 | 20.85 +/- 1.35 | 20 | 19-23 | 3 | 337 | 40 | 0 |
|  | CAST/EiJ 3 | 21.45 +/- 1.82 | 20 | 19-26 | 1 | 329 | 50 | 0 |
|  | CAST/EiJ 4 | 21.22 +/- 1.51 | 23 | 19-24 | 0 | 386 | 51 | 0 |
|  | CAST/EiJ 5 | 22.09 +/- 1.90 | 22 | 19-25 | 5 | 340 | 73 | 0 |
| **Total** |  | **21.28 +/- 1.64** | **105** | **19-26** | **15** | **1726** | **254** | **0** |
|  |  |  |  |  |  |  |  |  |
|  | C3H/HEJ 1230 | 23.08 +/- 1.59 | 40 | 19-27 | 5 | 589 | 164 | 2 |
|  | C3H/HEJ 1313 | 21.91 +/- 1.59 | 32 | 19-24 | 7 | 502 | 98 | 1 |
|  | C3H/HEJ 1314 | 21.85 +/- 2.10 | 39 | 19-26 | 8 | 608 | 112 | 0 |
|  | C3H/HEJ 1339 | 23.44 +/- 2.01 | 27 | 20-29 | 7 | 384 | 117 | 5 |
|  | C3H/HEJ 1340 | 23.97 +/- 1.64 | 29 | 21-27 | 2 | 403 | 146 | 0 |
|  | C3H/HEJ 1424 | 22.19 +/- 1.94 | 27 | 19-26 | 6 | 415 | 92 | 0 |
|  | C3H/HEJ 1425 | 22.73 +/- 1.58 | 15 | 20-26 | 1 | 228 | 55 | 1 |
| **Total** |  | **22.70 +/- 1.94** | **209** | **19-29** | **36** | **3129** | **794** | **9** |
|  |  |  |  |  |  |  |  |  |
|  | C57BL/6J 568 | 25.44 +/- 2.62 | 18 | 21-30 | 8 | 217 | 110 | 7 |
|  | C57BL/6J 673 | 24.35 +/- 1.31 | 20 | 22-27 | 6 | 266 | 103 | 5 |
|  | C57BL/6J 766 | 25.00 +/- 2.05 | 21 | 20-28 | 8 | 267 | 114 | 10 |
|  | C57BL/6J 771 | 25.75 +/- 2.38 | 16 | 22-30 | 5 | 191 | 103 | 5 |
|  | C57BL/6J 900 | 24.70 +/- 2.18 | 27 | 20-29 | 14 | 339 | 152 | 8 |
| **Total** |  | **24.99 +/- 2.15** | **102** | **20-30** | **41** | **1280** | **582** | **35** |

*E0, E1, E2, E3 values denote homologous pairs with 0, 1, 2, or 3 MLH1 foci.
