# Supplementary material for: Variation in Genome-Wide Levels of Meiotic Recombination Is Established at the Onset of Prophase in Mammalian Males
Source: PLoS Genet. 2014 Jan 30;10(1):e1004125. doi: 10.1371/journal.pgen.1004125 (PMC3907295; doi:10.1371/journal.pgen.1004125)
Supplement: Table S2 — Mean +/− S.D. RAD51 foci numbers for each animal and inbred strain. (DOCX) [file pgen.1004125.s002.docx]

Table S2: Mean +/- S.D. RAD51 foci numbers for each animal and inbred strain

|  | **Mouse** | **RAD51 Ave +/- SD** | **No. of Cells** | **Range** |
| --- | --- | --- | --- | --- |
|  | CAST/EiJ 1 | 155.45 +/- 23.92 | 20 | 124-206 |
|  | CAST/EiJ 2 | 154.00 +/- 14.58 | 23 | 130-185 |
|  | CAST/EiJ 3 | 166.80 +/- 18.23 | 20 | 135-202 |
|  | CAST/EiJ 4 | 176.42 +/- 13.82 | 19 | 152-200 |
|  | CAST/EiJ 5 | 164.47 +/- 11.12 | 17 | 136-184 |
| **Total** |  | **162.98 +/- 18.63** | **99** | **124-206** |
|  |  |  |  |  |
|  | C3H/HEJ 1425 | 165.00 +/- 15.01 | 9 | 150-191 |
|  | C3H/HEJ 1451 | 165.19 +/- 18.25 | 16 | 139-202 |
|  | C3H/HEJ 1452 | 169.50 +/- 17.92 | 10 | 143-197 |
|  | C3H/HEJ 1485 | 205.90 +/- 40.02 | 10 | 134-265 |
|  | C3H/HEJ 1501 | 188.44 +/- 24.81 | 25 | 136-240 |
| **Total** |  | **179.90 +/- 27.98** | **70** | **134-265** |
|  |  |  |  |  |
|  | C57BL/6J 568 | 187.69 +/- 22.21 | 16 | 148-239 |
|  | C57BL/6J 673 | 240.27 +/- 36.52 | 22 | 172-303 |
|  | C57BL/6J 766 | 221.38 +/- 24.52 | 24 | 178-260 |
|  | C57BL/6J 771 | 229.21 +/- 29.72 | 24 | 172-288 |
| **Total** |  | **222.13 +/- 33.78** | **86** | **148-303** |
