# Supplementary material for: Variation in Genome-Wide Levels of Meiotic Recombination Is Established at the Onset of Prophase in Mammalian Males
Source: PLoS Genet. 2014 Jan 30;10(1):e1004125. doi: 10.1371/journal.pgen.1004125 (PMC3907295; doi:10.1371/journal.pgen.1004125)
Supplement: Table S3 — Mean +/− S.D. DMC1 foci numbers for each animal and inbred strain. (DOCX) [file pgen.1004125.s003.docx]

Table S3: Mean +/- S.D. DMC1 foci numbers for each animal and inbred strain

|  | **Mouse** | **DMC1 Ave +/- SD** | **No. of Cells** | **Range** |
| --- | --- | --- | --- | --- |
|  | CAST/EiJ 100 | 119.71 +/- 16.12 | 7 | 100-139 |
|  | CAST/EiJ 68 | 116.96 +/- 16.04 | 25 | 92-156 |
|  | CAST/EiJ 69 | 122.72 +/- 17.61 | 32 | 95-160 |
|  | CAST/EiJ 79 | 113.86 +/- 11.61 | 7 | 100-134 |
| **Total** |  | **119.52 +/- 16.42** | **71** | **92-160** |
|  |  |  |  |  |
|  | C3H/HEJ 1560 | 147.94 +/- 17.02 | 18 | 119-177 |
|  | C3H/HEJ 1632 | 165.63 +/- 25.78 | 8 | 124-206 |
|  | C3H/HEJ 1635 | 148.54 +/- 17.09 | 13 | 125-192 |
|  | C3H/HEJ 1636 | 144.67 +/- 13.37 | 21 | 116-187 |
| **Total** |  | **149.28 +/- 18.08** | **60** | **116-206** |
|  |  |  |  |  |
|  | C57BL/6J 1003 | 178.83 +/- 22.76 | 29 | 135-242 |
|  | C57BL/6J 1383 | 179.24 +/- 15.83 | 37 | 141-217 |
|  | C57BL/6J 1386 | 149.71 +/- 19.22 | 7 | 126-185 |
|  | C57BL/6J 900 | 192.97 +/- 16.86 | 36 | 133-220 |
| **Total** |  | **181.77 +/- 21.06** | **109** | **126-242** |
