# Supplementary material for: Variation in Genome-Wide Levels of Meiotic Recombination Is Established at the Onset of Prophase in Mammalian Males
Source: PLoS Genet. 2014 Jan 30;10(1):e1004125. doi: 10.1371/journal.pgen.1004125 (PMC3907295; doi:10.1371/journal.pgen.1004125)
Supplement: Table S4 — Mean +/− S.D. MSH4 foci numbers for each animal and inbred strain. (DOCX) [file pgen.1004125.s004.docx]

Table S4: Mean +/- S.D. MSH4 foci numbers for each animal and inbred strain

|  | **Mouse** | **MSH4 Ave +/- SD** | **No. of Cells** | **Range** |
| --- | --- | --- | --- | --- |
|  | CAST/EiJ 1 | 103.76 +/- 12.54 | 17 | 88-125 |
|  | CAST/EiJ 2 | 105.42 +/- 16.94 | 12 | 59-125 |
|  | CAST/EiJ 3 | 115.45 +/- 9.30 | 11 | 101-133 |
|  | CAST/EiJ 4 | 107.85 +/- 8.52 | 13 | 97-123 |
|  | CAST/EiJ 5 | 94.36 +/- 6.33 | 11 | 87-106 |
| **Total** |  | **105.30 +/- 12.84** | **64** | **59-133** |
|  |  |  |  |  |
|  | C3H/HEJ 1457 | 114.54 +/- 16.13 | 13 | 81-135 |
|  | C3H/HEJ 1485 | 113.68 +/- 16.77 | 19 | 92-154 |
|  | C3H/HEJ 1491 | 95.44 +/- 12.73 | 9 | 75-119 |
|  | C3H/HEJ 1501 | 99.14 +/- 11.44 | 7 | 81-118 |
|  | C3H/HEJ 1561 | 112.13 +/- 11.89 | 16 | 85-126 |
|  | C3H/HEJ 1632 | 106.45 +/- 5.85 | 11 | 96-115 |
| **Total** |  | **108.89 +/- 14.75** | **75** | **75-154** |
|  |  |  |  |  |
|  | C57BL/6J 568 | 105.24 +/- 16.19 | 25 | 80 -154 |
|  | C57BL/6J 673 | 104.07 +/- 20.02 | 14 | 59 -147 |
|  | C57BL/6J 766 | 125.16 +/- 15.38 | 19 | 96-152 |
|  | C57BL/6J 771 | 116.14 +/- 11.44 | 21 | 91-137 |
| **Total** |  | **112.72 +/- 17.54** | **79** | **59-154** |
