# Supplementary material for: Variation in Genome-Wide Levels of Meiotic Recombination Is Established at the Onset of Prophase in Mammalian Males
Source: PLoS Genet. 2014 Jan 30;10(1):e1004125. doi: 10.1371/journal.pgen.1004125 (PMC3907295; doi:10.1371/journal.pgen.1004125)
Supplement: Table S5 — Mean +/− S.D. BLM foci numbers for each animal and inbred strain. (DOCX) [file pgen.1004125.s005.docx]

Table S5: Mean +/- S.D. BLM foci numbers for each animal and inbred strain

|  | **Mouse** | **BLM Ave +/- SD** | **No. of Cells** | **Range** |
| --- | --- | --- | --- | --- |
|  | CAST/EiJ 69 | 112.12 +/- 15.94 | 33 | 85-158 |
|  | CAST/EiJ 79 | 128.36 +/- 10.24 | 11 | 112-147 |
|  | CAST/EiJ 80 | 111.91 +/- 18.48 | 23 | 80-157 |
|  | CAST/EiJ 81 | 112.84 +/- 15.12 | 19 | 87-144 |
|  | CAST/EiJ 99 | 114.67 +/- 10.02 | 12 | 99-129 |
| **Total** |  | **114.35 +/- 15.87** | **98** | **80-158** |
|  |  |  |  |  |
|  | C3H/HEJ 1561 | 120.50 +/- 8.45 | 18 | 110-137 |
|  | C3H/HEJ 1632 | 120.00 +/- 14.60 | 8 | 105-143 |
|  | C3H/HEJ 1633 | 131.86 +/- 12.25 | 7 | 116-148 |
|  | C3H/HEJ 1634 | 113.55 +/- 7.30 | 20 | 101-125 |
|  | C3H/HEJ 1636 | 125.43 +/- 19.78 | 7 | 103-162 |
| **Total** |  | **120.02 +/- 12.35** | **60** | **101-162** |
|  |  |  |  |  |
|  | C57BL/6J 1003 | 144.56 +/- 22.74 | 18 | 110-192 |
|  | C57BL/6J 1300 | 130.55 +/- 20.38 | 20 | 98-169 |
|  | C57BL/6J 1386 | 166.75 +/- 29.19 | 20 | 122-225 |
|  | C57BL/6J 1907 | 142.08 +/- 12.79 | 13 | 115-159 |
| **Total** |  | **146.41 +/- 26.31** | **71** | **98-225** |
