# Supplementary material for: Variation in Genome-Wide Levels of Meiotic Recombination Is Established at the Onset of Prophase in Mammalian Males
Source: PLoS Genet. 2014 Jan 30;10(1):e1004125. doi: 10.1371/journal.pgen.1004125 (PMC3907295; doi:10.1371/journal.pgen.1004125)
Supplement: Table S6 — Mean +/− S.D. RAD51 foci numbers for Spo11+/− and Spo11+/+ males. (DOCX) [file pgen.1004125.s006.docx]

Table S6: Mean +/- S.D. RAD51 foci numbers for *Spo11^+/-^* and *Spo11^+/+^* males.

|  | **Mouse** | **RAD51 Ave +/- SD** | **No. of Cells** | **Range** |
| --- | --- | --- | --- | --- |
|  | SPO11-het 33 | 157.44 +/- 23.35 | 16 | 127-216 |
|  | SPO11-het 52 | 161.76 +/- 17.17 | 21 | 137-191 |
|  | SPO11-het 59 | 144.50 +/- 14.28 | 16 | 123-169 |
|  | SPO11-het 94 | 140.64 +/- 20.69 | 14 | 115-182 |
| **Total** |  | **152.19 +/- 20.55** | **67** | **115-216** |
|  |  |  |  |  |
|  | SPO11-wt 34 | 200.44 +/- 20.44 | 9 | 171-233 |
|  | SPO11-wt 51 | 203.71 +/- 22.97 | 14 | 169-247 |
|  | SPO11-wt 54 | 194.46 +/- 19.46 | 13 | 169-247 |
|  | SPO11-wt 58 | 202.60 +/- 23.54 | 15 | 169-258 |
| **Total** |  | **200.45 +/- 21.54** | **51** | **169-258** |
|  |  |  |  |  |
