# Supplementary material for: Variation in Genome-Wide Levels of Meiotic Recombination Is Established at the Onset of Prophase in Mammalian Males
Source: PLoS Genet. 2014 Jan 30;10(1):e1004125. doi: 10.1371/journal.pgen.1004125 (PMC3907295; doi:10.1371/journal.pgen.1004125)
Supplement: Table S7 — Mean +/− S.D. MLH1 foci numbers for Spo11+/− and Spo11+/+ males. (DOCX) [file pgen.1004125.s007.docx]

Table S7: Mean +/- S.D. MLH1 foci numbers for *Spo11^+/-^* and *Spo11^+/+^* males.

|  | **Mouse** | **MLH1 Ave +/- SD** | **No. of Cells** | **Range** | **E0*** | **E1*** | **E2*** | **E3*** |
| --- | --- | --- | --- | --- | --- | --- | --- | --- |
|  | SPO11-het 33 | 23.82 +/- 2.04 | 22 | 20-27 | 10 | 160 | 58 | 0 |
|  | SPO11-het 52 | 23.85 +/- 1.92 | 27 | 20-27 | 9 | 369 | 130 | 5 |
|  | SPO11-het 59 | 24.05 +/- 1.61 | 20 | 22-27 | 16 | 256 | 99 | 9 |
|  | SPO11-het 94 | 23.95 +/- 1.96 | 21 | 20-28 | 13 | 278 | 99 | 9 |
| **Total** |  | **23.91 +/- 1.86** | **105** | **20-28** | **48** | **1063** | **386** | **23** |
|  |  |  |  |  |  |  |  |  |
|  | SPO11-wt 34 | 23.86 +/- 1.96 | 21 | 21-27 | 6 | 234 | 83 | 0 |
|  | SPO11-wt 51 | 23.50+/- 1.58 | 26 | 21-26 | 7 | 365 | 120 | 2 |
|  | SPO11-wt 54 | 23.77 +/- 1.63 | 22 | 21-27 | 7 | 300 | 110 | 1 |
|  | SPO11-wt 58 | 24.00 +/- 1.37 | 17 | 22-27 | 11 | 219 | 90 | 3 |
| **Total** |  | **23.76 +/- 1.64** | **86** | **21-27** | **31** | **1118** | **403** | **6** |

*E0, E1, E2, E3 values denote homologous pairs with 0, 1, 2, or 3 MLH1 foci.
